# Impact of Race on Admission, Clinical Outcomes, and Disposition in Cholangiocarcinoma: Insights from the National Inpatient Database

**DOI:** 10.3390/diseases13070211

**Published:** 2025-07-04

**Authors:** Tijin A. Mathew, Teresa M. Varghese, Nithya Krishnakumaran, George M. Varghese, Khwaja S. Haq, Akshita Khosla, Rojymon Jacob, Gina Vaccaro

**Affiliations:** 1 Department of Internal Medicine, Southeast Health Dothan, Dothan, AL 36301, USA; 2 Department of Internal Medicine, Wellstar Spalding, Griffin, GA 30224, USA; 3 Department of Cardiovascular Medicine, Stanford University, Stanford, CA 94305, USA; 4 Department of Hematology and Oncology, Louisiana State University, New Orleans, LA 70112, USA; 5 Department of Radiation Oncology, The University of Mississippi Medical Center, Jackson, MS 39216, USA; 6 Department of Hematology Oncology, Tennessee Oncology, Nashville, TN 37203, USA

**Keywords:** cholangiocarcinoma, racial disparities, mortality, in-hospital complications

## Abstract

Background: Cholangiocarcinoma, malignancies arising from the intrahepatic and extrahepatic bile ducts, has increased in incidence in the United States over the past few decades. The reported incidence of cholangiocarcinomas is high, particularly in specific racial groups such as Asian and Pacific Islander patients. Race also significantly impacts disparities in healthcare utilization and clinical outcomes. Our study focused on the impact of race on admission, clinical outcomes, and disposition of cholangiocarcinoma. Methods: We performed a retrospective analysis of cholangiocarcinoma-related hospital admissions, using the National Inpatient Sample for the year 2022. Patients were stratified according to race into the following groups: White, African American, Hispanic, Asian or Pacific Islander, Native American, and Other. The data analysis was performed using STATA/BE version 18.5. Univariable and multivariable logistic regression models were applied to evaluate the relationship between race and clinical and healthcare utilization outcomes. Results: In 2022, 7479 hospitalizations were recorded for cholangiocarcinoma in the United States. Among these, 65.99% were White, 13.27% Hispanic, and 10.13% African American. There was a statistically significant difference in gender distribution across racial groups (*p* < 0.001), with males comprising the majority in all groups. Males outnumbered females in all racial groups except among the Hispanic group. Significant racial disparities in mortality were observed, with White patients showing a mortality rate of 6.69%, compared to higher rates among African American (9.76%), Native American (8.51%), and Asian or Pacific Islander (8.09%) patients, while Hispanic (5.04%) and Other (5.88%) groups had lower rates (*p* < 0.001). Conclusions: The study underscores the racial disparities among cholangiocarcinoma hospitalizations, with African American, Native American, and Asian patients facing disproportionately higher mortality and poorer in-hospital outcomes compared to White patients. This analysis highlights the healthcare strategies and policy reforms to promote equitable treatment by mitigating these disparities and to improve cholangiocarcinoma outcomes.

## 1. Introduction

Cholangiocarcinomas are cancers arising from the intrahepatic and extrahepatic bile ducts, accounting for 10–25% of all hepatobiliary malignancies [1,2]. Intrahepatic cholangiocarcinoma is rare, with approximately 2500 cases diagnosed annually in the United States [3,4]. The incidence of cholangiocarcinoma has increased in the past few decades [3,5]. Cholangiocarcinoma in the United States has been rising, especially for intrahepatic cholangiocarcinoma. Several comprehensive epidemiological studies reveal that from 2001 to 2017, the incidence of intrahepatic cholangiocarcinoma nearly doubled, with an annual percentage change (APC) ranging from about 3% to 9% [6,7]. Cholangiocarcinoma incidence rates are highest in Asian and Pacific Islander populations in the United States, followed by Hispanic populations, while non-Hispanic Black and White groups have lower rates [8,9,10].

The common essential risk factors for cholangiocarcinoma include diabetes, cirrhosis, viral hepatitis, and obesity. Less common risks include tobacco use, inflammatory bowel disease, host genetic polymorphisms, and alcohol consumption [11,12,13]. Other risk factors associated with cholangiocarcinoma are Opisthorchis viverrini or Clonorchis sinensis parasitic infections, primary sclerosing cholangitis, and bile duct cysts [13,14]. Cholangiocarcinoma portends a high risk of mortality. The aim of our study focused on the impact of race on admission, clinical outcomes, and disposition of cholangiocarcinoma.

## 2. Methods

### 2.1. Database—The National Inpatient Sample 2022

This study utilized the National Inpatient Sample (NIS), a widely recognized and publicly accessible database in the United States that compiles inpatient healthcare information from various payers. The NIS is an essential resource for large-scale research, providing detailed regional and national data on hospital utilization, patient access, healthcare costs, quality metrics, insurance status, demographic characteristics, and clinical outcomes [15,16]. Developed under the Healthcare Cost and Utilization Project (HCUP) by the Agency for Healthcare Research and Quality (AHRQ), the dataset is created through a partnership of federal, state, and private entities. It includes a stratified sample of about 20% of hospital discharges nationwide annually. According to the institutional guidelines at Southeast Health, research involving fully de-identified datasets like the NIS is exempt from Institutional Review Board (IRB) review. Therefore, this project was determined not to require IRB approval.

### 2.2. Study Population and Study Variable

The data from the 2022 National Inpatient Sample (NIS) were analyzed, and hospital admissions for adults (age > 18 years) with either a primary or secondary diagnosis of cholangiocarcinoma were identified using ICD-10-CM (International Classification of Diseases, Tenth Revision, Clinical Modification) codes. The ICD-10 codes for cholangiocarcinoma include C22.1 (Intrahepatic bile duct carcinoma), C24.0 (Extrahepatic bile duct carcinoma, C24.8 (Overlapping biliary duct carcinoma), and C24.9 (Unspecified biliary duct carcinoma). Racial groups, including White, African American, Hispanic, Asian or Pacific Islander, Native American, and Other (typically includes individuals who identify with races not explicitly listed, such as multiracial individuals, those from smaller racial groups, or those whose race was reported as “other” on hospital records), were recorded. Sociodemographic characteristics—such as age, gender, insurance status, and income quartile—and hospital-related factors, including facility size, geographic location, U.S. region, and teaching designation, were examined across these racial categories. The key clinical outcomes assessed in this study included in-hospital mortality, discharge disposition, total hospitalization costs, and length of stay.

### 2.3. Statistical Analysis

A cross-sectional study design was used, and data analysis was conducted using STATA/BE version 18.5. Chi-square tests were employed to examine associations between categorical variables, while independent *t*-tests were used to compare continuous variables. A *p*-value of less than 0.05 was considered statistically significant. Multivariable logistic regression models were applied to evaluate the relationship between race and various clinical and utilization outcomes. These outcomes included the type of hospital admission, discharge status, healthcare resource utilization, and complications such as mechanical ventilation, acute kidney injury, and sepsis. The analyses adjusted for potential confounding variables, which included obesity, sociodemographic factors, and hospital characteristics.

## 3. Results

### 3.1. Patient- and Hospital-Level Characteristics

In 2022, 7479 hospitalizations were recorded for cholangiocarcinoma. Of these, 53.6% were male, and 46.4% were female. A total of 65.99% of patients identified themselves as White, 13.27% as Hispanic, 10.13% as African American, 6.78% as Asian or Pacific Islander, and 3.81% as Native American or part of other minority groups.

The average age of the patient population was 68 years, with a 95% confidence interval (CI) ranging from 67.7 to 68.3. When looking at specific racial groups, the mean age was 66 years for African American and Hispanic patients. In comparison, the average ages were 69 years for White patients, 67 years for Asian or Pacific Islander patients, and 68 years for Native American patients. A greater proportion of African American (43.13%) and Hispanic (43.90%) patients were aged 64 years or younger compared to White patients (30.71%). Conversely, the majority of White (69.28%), Asian or Pacific Islander (64.49%), and Native American (65.95%) patients were aged 65 years or older. In contrast, only 56.86% of African American and 46.09% of Hispanic patients were in this older age group. (*p* < 0.001)

There was a statistically significant difference in gender distribution across racial groups (*p* < 0.001), with males comprising the majority in all groups, especially among Asian or Pacific Islander patients and Native American patients. However, in Hispanic populations, the females outnumbered the males. There were real socioeconomic disparities identified in the study population, with Asian or Pacific Islander (45.80%) and White groups (29.91%) overrepresented in higher income brackets, and African American (43.11%), Hispanic (31.73%), and Native American (47.73%) groups overrepresented in the lowest income bracket.

White (66.65%) and Native American (65.22%) are most likely to have Medicare, while Medicaid coverage was most common among Hispanic (21.99%), Asian or Pacific Islander (14.69%), and African American (13.20%) patients. Private insurance was relatively evenly distributed, and self-pay was most frequent among Hispanic patients (4.05%) and least common among Native American patients (*p* < 0.001). The racial distributions vary significantly by the U.S. hospital region, the South having the highest percentage of African American with 57.26%, the West the highest Hispanics with 39.17%, Asian or Pacific Islander (52.47%), and Native American (40.43%) patients, and all differences showing strong statistical significance (*p* < 0.001). Regardless of race and geography (urban vs. rural), most patients received care at teaching hospitals with a *p* < 0.001. The patient- and hospital-level characteristics are summarized in Table 1.

### 3.2. Primary Outcomes

**Figure 1:**
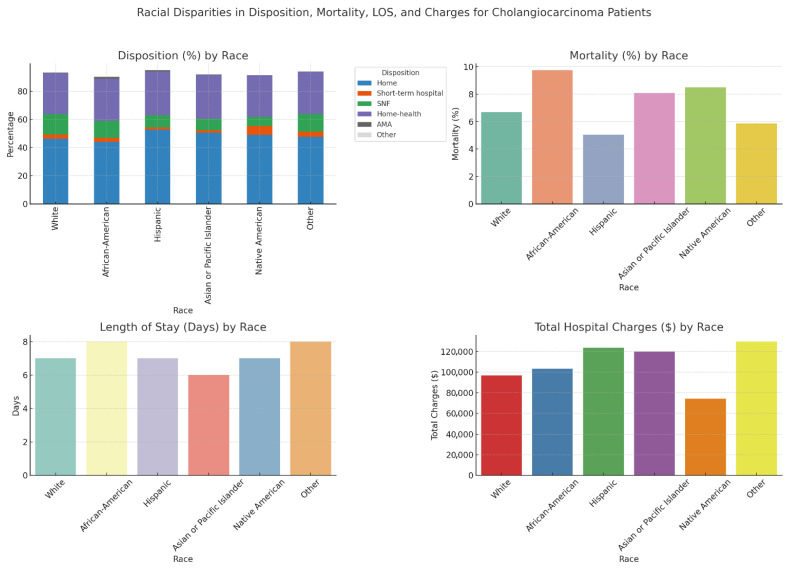
Racial inequities in admission and disposition outcomes and healthcare utilization among cholangiocarcinoma patients in the NIS 2022.

Among various racial groups, most patients were discharged to their homes, with 52.67% Hispanic patients and 44.06% African American patients. Home health care was the most common discharge disposition, accounting for 31.16% of Asian or Pacific Islander patients and 31.12% of Hispanic patients. A skilled nursing facility was the most frequent discharge destination for White patients at 14.28%, while it was the least common among Native American patients at 6.36%. The differences observed were statistically significant, with a *p*-value of less than 0.001. In the univariable analysis, without adjusting for confounders, the African American population had the highest mortality rate at 9.76%. Native American patients followed this at 8.51% and Asian or Pacific Islander patients at 8.09%. The Hispanic population had the lowest mortality rate at 5.04% (*p* < 0.001). The median length of stay varies significantly, with the highest length observed in the African American population and others with a mean length of stay of 8 days, and the least in Asian or Pacific Islander patients with 6 days. The total hospitalization charges also varied significantly, with the highest median total charges observed in other minor groups (USD 129,606) and Hispanic population (USD 123,703), and the lowest among Native American patients (USD 74,064) (*p* < 0.001). Most hospitalizations across all racial groups were for non-elective admissions (*p* < 0.001). The racial inequities in admission and disposition outcomes and healthcare utilization among cholangiocarcinoma patients are summarized in Table 2 and Figure 1.

Multivariable logistic regression analysis was also performed after adjusting for confounders and White as the reference group. Compared to White patients, African American (AOR 0.70, 95% CI: 0.54–0.89, *p* = 0.004), Hispanic (AOR 0.62, 95% CI: 0.50–0.77, *p* < 0.001), and Asian or Pacific Islander patients (AOR 0.67, 95% CI: 0.51–0.89, *p* = 0.006) had significantly lower odds of elective admission, while Native American patients had similar odds (AOR 1.16, 95% CI: 0.53–2.54, *p* = 0.38). The odds of death were as follows: African American patients had significantly higher odds (AOR 1.44, 95% CI: 1.10–1.90, *p* = 0.007) and Hispanic patients had lower odds (AOR 0.70, 95% CI: 0.51–0.96, *p* = 0.02) compared to White patients, with no significant difference observed for Asian or Pacific Islander or Native American patients. The multivariable regression analysis affecting racial inequities in admission and mortality of cholangiocarcinoma patients is summarized in Table 3.

### 3.3. In-Hospital Complications

**Figure 2:**
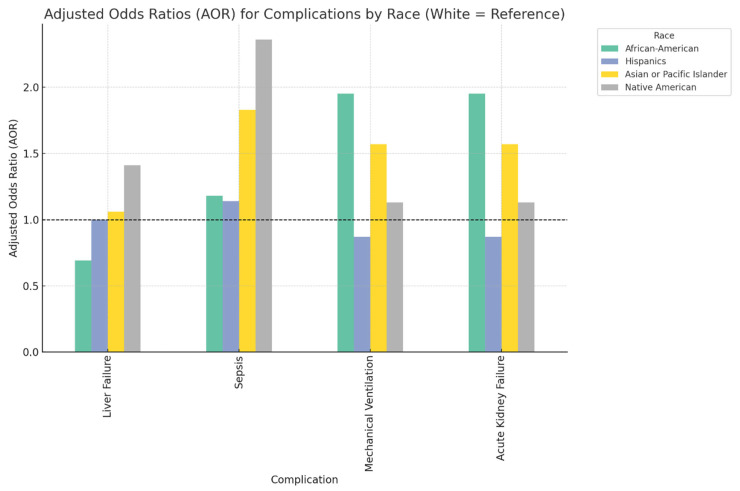
Bar chart showing racial differences in the complications associated with cholangiocarcinoma patients in the NIS 2022.

Compared to White patients, African American patients demonstrated a significantly lower likelihood of developing liver failure during hospitalization (AOR 0.69, 95% CI: 0.49–0.97, *p* = 0.03). However, they were nearly twice as likely to require mechanical ventilation (AOR 1.95, 95% CI: 1.30–2.92, *p* < 0.001) and to experience acute kidney failure (AOR 1.95, 95% CI: 1.30–2.92, *p* < 0.001). In contrast, Asian or Pacific Islander patients had a markedly increased risk of developing sepsis (AOR 1.83, 95% CI: 1.41–2.36, *p* < 0.001), as did Native American patients (AOR 2.36, 95% CI: 1.12–5.02, *p* = 0.02), when compared to White patients. Notably, there were no statistically significant differences in the risk of these in-hospital complications for Hispanic patients relative to White patients. The racial differences in the complications associated with cholangiocarcinoma are summarized in Figure 2 and Table 4.

## 4. Discussion

The results of this study underscore the presence of notable racial disparities among patients hospitalized with cholangiocarcinoma in the United States, as evidenced by the analysis of 2022 National Inpatient Sample (NIS) data. The investigation reveals that hospitalization patterns, clinical outcomes, and healthcare utilization exhibited significant variability across distinct racial cohorts. The age distribution of cholangiocarcinoma indicates that the incidence of this cancer increases with age across all racial groups, with the highest rates observed among older adults. Interestingly, while the incidence of cholangiocarcinoma is nearly twice as high in Asian patients compared to White patients [8,17,18], our analysis showed that the overall distribution was higher in the White older population, followed by Native American and Asian patients.

The national data indicate that mortality rates associated with cholangiocarcinoma have risen across all racial groups [3,4]. Our analysis focused on all racial groups, comparing them to previous smaller studies. Notably, the highest increase occurred among African American patients, with a 45% rise in mortality from 2000 to 2014; a 22% increase followed this in mortality rates among Asian patients, and a 20% increase among White patients during the same period [19]. In our study, we found that African American patients had significantly worse in-hospital outcomes compared to White patients, followed by Native American and Asian patients. These findings highlight persistent disparities in cholangiocarcinoma outcomes and raise essential questions about the underlying systemic factors driving these inequities across groups.

The disparities observed in cholangiocarcinoma outcomes across racial groups are likely influenced by a combination of socioeconomic and systemic factors [9,20]. Our analysis was consistent with national data demonstrating higher mortality rates among minority populations [9,21]. Socioeconomic status, insurance coverage, and access to quality post-acute care are critical determinants of outcomes. Prior research has shown that Black patients are less likely to receive surgical intervention for cholangiocarcinoma, and that lack of private insurance and lower income are associated with decreased overall survival [9]. This evidence highlights the urgent need for targeted interventions that address social determinants of health and improve access to high-quality care for minority patients with cholangiocarcinoma. Additionally, it emphasizes the importance of including diverse populations in research and clinical trials to inform equitable treatment guidelines.

Significant racial disparities were observed in the in-hospital complications of cholangiocarcinoma patients. African American patients were less likely to develop acute liver failure compared to White populations; however, the risk of mechanical ventilation and development of acute kidney failure was nearly doubled in these populations. These observations coincide with previous studies that reported higher adverse outcomes and resource utilization among African American patients with cholangiocarcinoma [21,22,23].

Discharge disposition may reflect and can influence disparities in healthcare outcomes. Our data indicates that White patients, who are often in higher income brackets, are more likely to be discharged to skilled nursing facilities. These facilities typically offer more comprehensive post-acute care. In contrast, African American, Hispanic, and Native American patients, who are disproportionately found in lower income brackets, are more frequently discharged to home or with home health services. Previous research has shown that access to higher-quality post-discharge care, such as that provided by skilled nursing facilities, can lead to improved recovery and long-term outcomes [24,25]. Conversely, limited access to such care may contribute to higher readmission rates and poorer survival outcomes [24,25].

As we analyze these results, it is vital to consider the broader implications for healthcare delivery and policy to improve the quality of care for marginalized racial groups. Understanding such health outcome variations among racial groups is crucial for developing tailored diagnostic and management protocols, ensuring equitable treatment efficacy.

## 5. Limitations of the Study

The NIS database does not contain comprehensive patient-level information, including details about medications, laboratory results, and tumor features, which could significantly affect outcomes in cholangiocarcinoma but were not factored into our analysis [26,27]. Consequently, we could not rule out these factors as potential confounders in the outcomes we examined. Additionally, since the NIS relies on administrative coding, there is a risk of misclassification or coding inaccuracies, which may impact the validity of the diagnoses and complication rates reported [26,27]. The cross-sectional nature of our study also constrains our capacity to establish causal links between race, socioeconomic status, and patient outcomes; we can only discuss the associations evident in the data [28]. Although we attempted to adjust for various confounding factors, unmeasured variables may still have influenced the results.

Furthermore, since the NIS data is limited to hospitalized patients in the United States, our findings may not apply to populations in other countries or individuals who did not require hospitalization [27]. The NIS database also does not track long-term outcomes or rates of readmission, which limits our ability to analyze long-term survival or the effects of repeated hospital stays [28]. Nevertheless, the significant disparities related to race and socioeconomic status identified in our research highlight the critical need for further studies and targeted interventions aimed at addressing inequalities in the care and outcomes of cholangiocarcinoma patients [29,30].

The cholangiocarcinoma diagnoses in the NIS are categorized as intrahepatic, extrahepatic, or unspecified bile duct cancer. Many patients fall under the “unspecified” category, limiting our ability to conduct reliable subgroup analyses without introducing selection bias. Therefore, to preserve the generalizability of our findings, we presented outcomes using a broader classification of cholangiocarcinoma. The NIS captures both primary and secondary diagnoses, and due to the nature of administrative coding, we cannot definitively determine the principal reason for hospitalization in all cases. Patients may be admitted for treatment complications, unrelated comorbidities, or disease progression. Third, the NIS lacks granular clinical data such as TNM staging, tumor pathology, performance status, and detailed treatment information (e.g., chemotherapy, radiation, or surgery). As a result, we were unable to evaluate outcomes based on cancer stage or treatment modality. These limitations should be considered when interpreting our findings, and they highlight the need for future research using more detailed clinical databases.

## 6. Conclusions

In conclusion, this study highlights pronounced racial and socioeconomic disparities in the hospitalization outcomes of patients with cholangiocarcinoma. African American, Native American, and Asian patients face disproportionately higher mortality and poorer in-hospital outcomes compared to their White counterparts. Moreover, factors such as lower income and less access to skilled post-discharge care may contribute to these adverse outcomes among minority populations. These findings emphasize the critical need for focused healthcare strategies and policy reforms aimed at reducing these gaps and promoting equitable treatment and support for all individuals affected by cholangiocarcinoma.

## Figures and Tables

**Table 1:** Patient- and hospital-level characteristics of hospitalized cholangiocarcinoma patients stratified by race in the NIS 2022.

Variables	White [4936] (%)	African American [758] (%)	Hispanic [993] (%)	Asian or Pacific Islander [507] (%)	Native American [47] (%)	Other [238] (%)	*p*-Value
Age							
Mean age (years)	69	66	66	67	68	64	
Age <= 64 years	30.71	43.13	43.90	35.50	34.04	46.63	<0.001
Age >= 65 years	69.28	56.86	46.09	64.49	65.95	53.36	
Gender							
Male (%)	54.32	50.40	48.44	59.37	59.57	57.14	<0.001
Female (%)	45.68	49.60	51.56	40.63	40.43	42.86	
Median household income national quartiles							
Quartile 1 (0–25th percentile) *	19.08	43.11	31.73	7.20	47.73	17.09	<0.001
Quartile 2 (26–50th percentile) **	24.14	21.29	20.88	19.60	11.36	17.09	
Quartile 3 (51st–75th percentile) ***	26.86	21.02	27.94	27.40	29.55	27.78	
Quartile 4 (76th–100th percentile) ****	29.91	14.59	19.45	45.80	11.36	38.03	
Insurance type							
Medicare	66.65	55.92	51.87	54.93	65.22	45.02	<0.001
Medicaid	5.41	13.20	21.99	14.69	8.70	22.08	
Private, including Health Maintenance Organization	26.86	28.16	22.10	28.37	26.09	30.30	
Self-pay	1.08	2.72	4.05	2.01	0.00	2.60	
Hospital region							
Northeast	23.26	17.15	15.91	18.74	2.13	31.09	<0.001
Midwest	25.83	18.47	9.26	10.65	23.40	12.61	
South	33.35	57.26	35.65	18.15	34.04	30.67	
West	17.56	7.12	39.17	52.47	40.43	25.63	
Teaching status							
Non-teaching	16.47	11.61	9.97	14.20	21.28	14.29	<0.001
Teaching	83.53	88.39	90.03	85.80	78.72	85.71	
Hospital bed size							
Small	15.82	15.44	11.38	10.06	25.53	11.76	<0.001
Medium	24.82	21.64	23.36	23.67	23.40	21.01	
Large	59.36	62.93	65.26	66.27	51.06	67.23	

(* Quartile 1 = USD 1–55,999, ** Quartile 2 = USD 56,000–70,999, *** Quartile 3 = USD 71,000–93,999, **** Quartile 4 = USD 94,000 or more).

**Table 2:** Racial inequities in admission and disposition outcomes and healthcare utilization among cholangiocarcinoma patients from NIS 2022.

Outcomes	White [4936] (%)	African American [758] (%)	Hispanic [993] (%)	Asian or Pacific Islander [507] (%)	Native American [47] (%)	Other [238] (%)	*p*-Value
Patient Disposition							
Home	46.25	44.06	52.67	50.49	48.94	47.48	<0.001
Short-Term Hospital	3.06	2.77	1.51	1.78	6.38	3.78	
Skilled Nursing Facility	14.28	12.14	8.76	8.09	6.36	12.61	
Home Health	29.19	29.82	31.12	31.16	29.79	29.83	
Against Medical Advice	0.47	1.45	0.91	0.39	0.00	0.42	
Other	0.06	0.00	0.00	0.00	0.00	0.00	
Mortality	6.69	9.76	5.04	8.09	8.51	5.88	<0.001
Length of Stay (Days)	7	8	7	6	7	8	<0.001
Total Charge (USD)	96,596	103,490	123,703	119,909	74,064	129,606	<0.00

**Table 3:** Multivariable regression analysis affecting racial inequities in admission and mortality of cholangiocarcinoma patients in the NIS 2022.

	Elective vs. Non-Elective Admission AOR * [95% CI, *p*-Value]	Mortality AOR * [95% CI, *p*-Value]
White [4936]	Reference	Reference
African American [758]	0.70 (0.54–0.89, 0.004)	1.44 (1.10–1.90, 0.007)
Hispanic [993]	0.62 (0.50–0.77, 0.00)	0.70 (0.51–0.96, 0.02)
Asian or Pacific Islander [507]	0.67 (0.51–0.89, 0.00)	1.2 (0.85–1.7, 0.28)
Native American [47]	1.16 (0.53–2.54, 0.38)	1.4 (0.47–1.47, 0.53)

* Adjusted Odds Ratio (AOR) (for confounders: sociodemographic, hospital characteristics, the Charlson comorbidity index, and other cardiac risk factors, including tobacco abuse, hypertension, hyperlipidemia, diabetes, cannabis use, and obesity).

**Table 4:** Multivariable regression analysis of racial differences in the occurrence of various complications associated with cholangiocarcinoma patients in the NIS 2022.

Complications	White	African American AOR * [95% CI, *p*-Value]	Hispanics AOR * [95% CI, *p*-Value]	Asian or Pacific Islander AOR * [95% CI, *p*-Value]	Native American AOR * [95% CI, *p*-Value]
Liver Failure	Reference	0.69 (0.49–0.97, 0.03)	1.00 (0.76–1.32, 0.96)	1.06 (0.73–1.53, 0.33)	1.41 (0.89–1.53, 0.74)
Sepsis	Reference	1.18 (0.92–1.51, 0.16)	1.14 (0.91–1.43, 0.23)	1.83 (1.41–2.36, 0.00)	2.36 (1.12–2.56, 0.02)
Mechanical Ventilation	Reference	1.95 (1.3–2.92, 0.0010)	0.87 (0.74–1.4, 0.59)	1.57 (0.92–1.72, 0.07)	1.13 (0.87–1.36, 0.13)
Acute Kidney Failure	Reference	1.95 (1.30–2.92, 0.00)	0.87 (0.59–1.40, 0.59)	1.57 (0.95–1.42, 0.07)	1.13 (0.96–1.43, 0.89)

* Adjusted Odds Ratio (AOR) (for confounders: sociodemographic, hospital characteristics, the Charlson comorbidity index, and other cardiac risk factors, including tobacco abuse, hypertension, hyperlipidemia, diabetes, cannabis use, and obesity).

## Data Availability

Data for this study are derived from public domain resources. The findings of these studies are corroborated by data from the HCUP, which researchers can access through a standard application process and a signed data use agreement. The authors note that they did not receive special access to the HCUP data used in this study (covering 2022). They paid the necessary fee to obtain the NIS data, as specified in the HCUP Central Distributor’s fee schedule, which manages purchase requests and data use agreements (DUAs) for all users (https://www.hcup-us.ahrq.gov/tech_assist/centdist.jsp, accessed on 19 December 2024). Researchers wishing to access HCUP databases must complete the online HCUP DUA (https://www.hcup-us.ahrq.gov/tech_assist/dua.jsp, accessed on 19 December 2024) and agree to its terms. Additional details on how to apply for HCUP database purchases are available at ([https://www.distributor.hcup-us.ahrq.gov], accessed on 19 December 2024), and the link for the NIS dataset can be found at www.hcup-us.ahrq.gov/nisoverview.jsp, accessed on 19 December 2024.
